# Cost-utility analysis of different treatments for post-traumatic stress disorder in sexually abused children

**DOI:** 10.1186/1753-2000-6-15

**Published:** 2012-04-10

**Authors:** Elena Gospodarevskaya, Leonie Segal

**Affiliations:** 1 Liverpool School of Tropical Medicine, Clinical Research Group, Pembroke Place, Liverpool L3 5QA, UK; 2 Division of Health Sciences, University of South Australia, Campus East, North Terrace, Adelaide, SA, Australia

## Abstract

**Background:**

Post-traumatic stress disorder (PTSD) is diagnosed in 20% to 53% of sexually abused children and adolescents. Living with PTSD is associated with a loss of health-related quality of life. Based on the best available evidence, the NICE Guideline for PTSD in children and adolescents recommends cognitive behavioural therapy (TF-CBT) over non-directive counselling as a more efficacious treatment.

**Methods:**

A modelled economic evaluation conducted from the Australian mental health care system perspective estimates incremental costs and Quality Adjusted Life Years (QALYs) of TF-CBT, TF-CBT combined with selective serotonin reuptake inhibitor (SSRI), and non-directive counselling. The "no treatment" alternative is included as a comparator. The first part of the model consists of a decision tree corresponding to 12 month follow-up outcomes observed in clinical trials. The second part consists of a 30 year Markov model representing the slow process of recovery in non-respondents and the untreated population yielding estimates of long-term quality-adjusted survival and costs. Data from the 2007 Australian Mental Health Survey was used to populate the decision analytic model.

**Results:**

In the base-case and sensitivity analyses, incremental cost-effectiveness ratios (ICERs) for all three active treatment alternatives remained less than A$7,000 per QALY gained. The base-case results indicated that non-directive counselling is dominated by TF-CBT and TF-CBT + SSRI, and that efficiency gain can be achieved by allocating more resources toward these therapies. However, this result was sensitive to variation in the clinical effectiveness parameters with non-directive counselling dominating TF-CBT and TF-CBT + SSRI under certain assumptions. The base-case results also suggest that TF-CBT + SSRI is more cost-effective than TF-CBT.

**Conclusion:**

Even after accounting for uncertainty in parameter estimates, the results of the modelled economic evaluation demonstrated that all psychotherapy treatments for PTSD in sexually abused children have a favourable ICER relative to no treatment. The results also highlighted the loss of quality of life in children who do not receive any psychotherapy. Results of the base-case analysis suggest that TF-CBT + SSRI is more cost-effective than TF-CBT alone, however, considering the uncertainty associated with prescribing SSRIs to children and adolescents, clinicians and parents may exercise some caution in choosing this treatment alternative.

## Background

It is estimated that 5-10% of girls and 1-5% of boys in high income countries are exposed to penetrative sexual abuse during childhood, with even higher prevalence rates if any form of sexual abuse is included [1]. Although between 1/2 and 2/3 of sexually abused symptomatic children improve over time [2], mental health consequences can be debilitating and the process of recovery may take many years and even result in premature mortality [3]. Post-traumatic stress disorder (PTSD) is frequently observed in sexually abused children who are often diagnosed with other mental health co-morbidities (e.g. anxiety, depression). PTSD and co-morbid depression are associated with an increased risk of suicide [4]. Studies conducted in the US population of sexually abused children reported the prevalence of PTSD ranging between 20% and 53% [2,5-7].

PTSD is characterised by symptoms lasting more than one month following an extremely traumatic event which the person experienced or witnessed (e.g. combat, terrorist attack or natural disaster). PTSD was first introduced in the Diagnostic and Statistical Manual of Mental Health Disorders in 1980 [8]. In 2000, childhood sexual abuse was recognised as a qualifying traumatic event [9], which typically results in intense fear, helplessness or horror. Three clusters of symptoms are associated with PTSD: re-experiencing the traumatic event, avoidance or emotional numbing and hyper-arousal. Re-experiencing may present in one or more of the following ways: intrusive recollections; recurring nightmares; acting or feeling as if the event were recurring; distress when reminded of the event; or physiological reactivity when reminded of the event. Children may also re-experience the traumatic event in the form of trauma-thematic spontaneous play. Symptoms of avoidance involve efforts to avoid thoughts, feelings, activities, places or people that arouse recollections of the event, inability to recall aspects of the trauma, and diminished interest or participation in significant events. In children, avoidance may lead to a restricted lifestyle, refusal to separate from parents and difficulty experiencing tender or loving feelings. Adolescents may resort to drug and alcohol abuse and demonstrate a foreshortened view of the future, being unable to envisage growing to maturity and having a long and fulfilling life. Symptoms of increased arousal involve difficulty falling or staying asleep; irritability or outbursts of anger; difficulty concentrating; hyper-vigilance (e.g. excessive checking of locks in the home and over-concern about health and welfare of parents); or an exaggerated startle response [10]. To be diagnosed with PTSD, children must exhibit at least one re-experiencing symptom, three avoidance/numbing symptoms, and two increased arousal symptoms [9].

The high prevalence of PTSD associated with sexual abuse led to the development of specialist psychotherapeutic treatments for which a reduction of PTSD symptoms is the primary outcome. In 2005, the UK National Institute for Health and Clinical Excellence (NICE) commissioned the National Collaborating Centre for Mental Health to produce a Clinical Practice Guideline for Management of PTSD in Adults and Children. Development of this PTSD Guideline included a systematic assessment of eight randomised controlled trials (RCTs) involving children with substantiated contact sexual abuse [11-19]. On the basis of available evidence the PTSD Guideline recommended 8-12 individual weekly Trauma-Focused Cognitive Behavioural Therapy (TF-CBT) sessions with the child over non-directive supportive counselling or standard community treatment for treating PTSD in children and adolescents [20]. There was insufficient evidence to support recommendation of other types of treatment such as play therapy or art therapy [20].

TF-CBT is a flexible component-based manualised treatment that typically includes relaxation skills, affective regulation skills, cognitive coping skills, trauma narrative and cognitive processing of the traumatic events, psychoeducation and parenting skills [21]. The core principle of TF-CBT is the "gradual exposure" of the child to the child's traumatic experience, where the intensity of the exposure incrementally and systematically increases throughout the treatment process. Non-directive supportive counselling typically includes establishing a trusting therapeutic relationship by providing active listening, reflection, accurate empathy, encouragement to talk about feelings and belief in the child's and parent's ability to develop positive coping strategies for abuse-related difficulties. Unlike TF-CBT, non-directive counselling is not a manualised treatment. It is primarily non-advisory but may include some elements of psycho-education about stress reaction and normalisation of PTSD symptoms [20].

In addition to recommendations regarding the content of therapy, the PTSD Guideline reviewed evidence and provided guidance regarding the modality of treatment. Although parental participation was not associated with additional PTSD-related clinical benefit for a child in the short term [16], the Guideline acknowledged the importance of parental reactions to the successful treatment of PTSD. Participating in treatment may reduce the level of anxiety in parents and carers and also improves their confidence and parenting practices, which may benefit the child over the long term [22]. However, the Guideline advised against treatment modalities involving only parents.

Depression is the most commonly observed co-morbidity in persons diagnosed with PTSD [23] and the association between these conditions was extensively researched (see Methods section below). The NICE guideline for treatment of depression in children and young people was consistent with the PTSD Guideline in its recommendation of CBT and supportive non-directive therapy as the treatment of first choice for depression in children and adolescents [24]. However, the Depression Guideline differs from the PTSD Guideline in relation to the use of pharmacotherapy in treatment of mental health conditions in children and adolescents. The Depression Guideline endorsed the use of fluoxetine in treatment of moderate to severe depression and the broader range of selective serotonin reuptake inhibitors (SSRIs) such as sertraline, citalopram, and paroxetine for depression unresponsive to psychotherapy. Pharmacotherapy is to be provided along with psychological therapy and the patients are to be monitored carefully for the appearance of suicidal behaviour. In contrast, the PTSD Guideline rejected the practice of "off-label" prescribing of psychotropic drugs for children [25-27], citing evidence of increased suicidal ideations and behaviour in young people who were taking SSRIs [28-30]. Currently, no SSRIs are approved for the treatment of PTSD in the USA paediatric population, however numerous authors have addressed the question of whether it is safe to use SSRIs in children. For example, a meta-analysis of 15 antidepressant trials [31] found no statistically significant difference in suicidal thoughts and behaviours between patients receiving antidepressants and those receiving placebo for depression. The authors concluded that the benefits of antidepressants appear to be much greater than risks from suicidal ideation and behaviour in depressed children and adolescents [31,32]. In relation to the study population of children and adolescents with PTSD secondary to sexual abuse, the apparent benefit of adding SSRI to psychotherapy was demonstrated in a small-size double-blind RCT in 10- to 17-year olds. The study compared outcomes of the intervention group assigned to 12 weekly individual TF-CBT sessions and SSRI (sertraline) with outcomes of the control group assigned to TF-CBT and placebo [33]. The number of children no longer meeting the full PTSD criteria (treatment responders) was higher in the TF-CBT and sertraline group, although the trial was underpowered to detect a statistically significant difference. All children with co-morbid depression (58% in each group) were among the treatment responders. The two groups showed no significant difference in measures of suicidal ideation at any observation point during the study.

To summarise, it appears that there is clinical evidence supporting the following treatments available to sexually abused children and adolescents who met all or most of diagnostic criteria for PTSD.

• Individual TF-CBT sessions with the child alone. This manualised treatment involves 12 sessions of 45 minutes duration provided on a weekly basis [12-16,18]. There is some limited evidence that a variation of TF-CBT, called Eye Movement Desensitization and Reprocessing treatment, which is also based on the concept of gradual exposure of sexually abused children to their traumatic experience is equally effective in treatment of PTSD as the standard TF-CBT [17].

• A combination therapy involving 12 individual parallel 45 min TF-CBT sessions with the child and non-abusive parent and SSRI [33].

• Twelve individual non-directive supportive counselling sessions of 45 min duration (used as a control group in some RCTs [12-15]).

CBT and individual non-directive supportive counselling are recommended as the first line treatment of depression in children and adolescents [24]; for severe depression a combination of psychiatric treatment and SSRIs can be considered. SSRIs (and sertraline in particular) are recommended as the second line treatment for those who do not respond to TF-CBT or non-directive counselling.

Although there was no experimental study that included all three of the recommended treatment alternatives (TF-CBT, TF-CBT + SSRI, and non-directive supportive counselling) in a single RCT, their comparative effectiveness in terms of the proportion of treatment responders (i.e. children who no longer meet the PTSD diagnostic criteria at the end of treatment) can be assessed using the method of indirect comparisons [34]. Allocating limited health care resources to the most cost-effective PTSD treatments would affect the balance of health benefits and costs for society; however there is a paucity of evidence regarding the cost-effectiveness of treatments in child and adolescent mental health [35]. Economic evaluation can assist by comparing costs and outcomes of different treatments and identifying those treatments with the lowest cost per unit of health gain [36]. Shifting resources away from services that are high cost per unit of health gain to those with a low cost per unit of health gain would increase the total health and wellbeing of society.

This paper employs a method of modelled economic evaluation to undertake a cost-utility analysis of different treatments for PTSD (individual TF-CBT with child; a combined treatment involving TF-CBT with child and pharmacotherapy (SSRI), and non-directive supportive counselling) versus a "no treatment" comparator. The "no treatment" comparator is routinely used in modelled economic evaluations, however in this particular analysis it acquires a real practical interpretation because not all sexually abused children with mental health problems are identified and subsequently treated. Cost-utility analysis produces incremental cost effectiveness ratios (ICER) comparing costs and outcomes of each of these treatments against a "no treatment" comparator and each other. The outcomes are expressed in quality-adjusted life years (QALYs), the measure of health that combines the effects of disease upon morbidity (e.g. the presence of PTSD and/or depression) and mortality (e.g. suicides in adolescents with a history of sexual abuse). The base-case analysis is conducted from the perspective of the Australian mental health system and does not assume any particular distribution of children across the treatment alternatives.

## Methods

### Outline of the economic model

The modelled economic evaluation of different treatments for sexually abused children with PTSD consists of two parts. The first part is a decision tree that models the costs and effects corresponding to the post-treatment and 12 month follow-up outcomes reported in RCTs of clinical interventions (TF-CBT, TF-CBT in combination with SSRI, and non-directive supportive counselling) and a no treatment comparator [15,16,33]. In these trials clinical effectiveness was reported in terms of proportions of treatment responders (i.e. children who no longer met DSM-IV diagnostic criteria for PTSD). The clinical effectiveness estimates were used to determine what proportions of the baseline cohort is assigned to "PTSD", "PTSD + depression" or "no PTSD/no Depression" health states at the end of 12 months. The second part of the model is a Markov process that calculates the long-term costs and outcomes in sexually abused children with PTSD or PTSD and depression over the next 30 year time interval.

The purpose of the decision tree is to model the treatment effect observed in the RCTs that inform the economic evaluation [15,16,33]. For each of the three active treatments and the no treatment alternative, the decision-tree tracks the proportion of the model cohort reaching the following health states at the end of 12 months: a) remission from PTSD (and depression if present at the baseline), and b) still meeting full diagnostic criteria for PTSD (and depression if present at the baseline).

**Figure 1:**
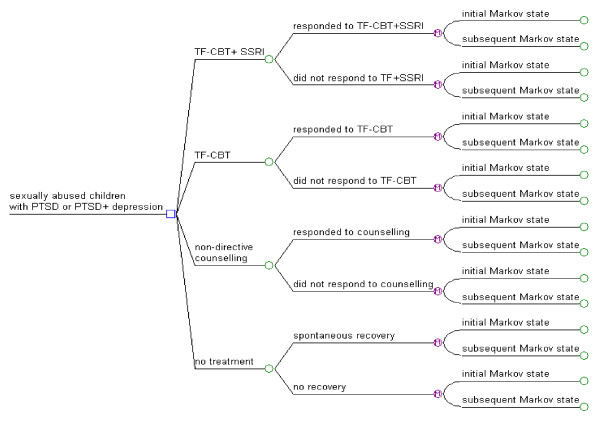
**Structure of the decision tree part of the modelled economic evaluation with 12 month time horizon**. PTSD - Post traumatic stress disorder; TF-CBT - Trauma-Focused Cognitive Behavioural Therapy; SSRI - Selective Serotonin Reuptake Inhibitor.

A schematic representation of the base-case model structure for the decision tree is shown in Figure 1. The model differentiates between responders and the small proportion of apparent non-responders who may experience a delayed treatment response [16,18]. For delayed responders, treatment response was assumed to occur 3 months after completion of 12 sessions (i.e. at mid-point of the 12 month time interval modelled with the decision tree). Delayed responders were assigned health benefits for 6 out of 12 months, while those who were no longer meeting PTSD diagnostic criteria at the post-treatment assessment were assigned health benefits for 9 out of 12 months. The rest of the treatment non-responders who at the baseline were diagnosed with PTSD or PTSD and depression were assumed to remain in these respective health states at the end of the 12 month time interval.

The objective of the Markov model is to estimate the long-term health and cost consequences for the model cohort over the next 30 years (i.e. the surviving proportion of cohort would be 41 years old at the end of the last Markov cycle). The model time horizon includes the age interval corresponding to the increase in the rates of suicide, which occurs between 20 to 34 years of age in males and between 24 to 29 years of age in females [37] and captures all health benefits associated with PTSD treatment and gradual spontaneous recovery from PTSD which is observed in about 2/3 of the baseline cohort by the age of 40. Consistent with the USA epidemiological evidence, the remaining part of the cohort (34%) is assumed not to recover from the PTSD associated with childhood sexual abuse [38].

Health benefits are measured in QALYs, which are defined as the product of life years and a preference-based index of quality of life (utility weight). Utility weights reflect subjective valuations of the relative worth of different health states where "1" represents perfect health and "0" represents death. Health states perceived as worse than death are allowed and carry a negative value. Utility weights exhibit equal interval properties and an equivalence with life years (e.g. a reduction of 0.2 quality of life utility score over 5 years is equivalent to the loss of 1 life year). Utility weights can be directly obtained from patients or the general public using measurement techniques such as the time trade-off or standard gamble [36]. Alternatively, off-the-shelf weights from questionnaires such as the Assessment of Quality of Life - AQoL [39] or European Quality of Life five dimensions - EQ-5D [40] can be applied to the population of interest. Total QALYs are then estimated by aggregating the utility-adjusted time intervals that patients spent in each subsequent health state. In estimating long-term costs and health consequences, it is assumed that the difference in effectiveness between the treatments only relates to the first 12 months for which the post-treatment and follow-up effectiveness outcomes are available. Subsequently the probabilities and payoffs (i.e. costs and utilities) of recurrent mental health problems (e.g. depression observed after the 12 month interval or the ongoing spontaneous recovery from PTSD experienced by a proportion of the cohort over the next 30 years) are independent from the evaluated treatments. Estimates of the expected long-term survival and costs are therefore conditional only on each patient's health state at the end of the 12 months.

**Figure 2:**
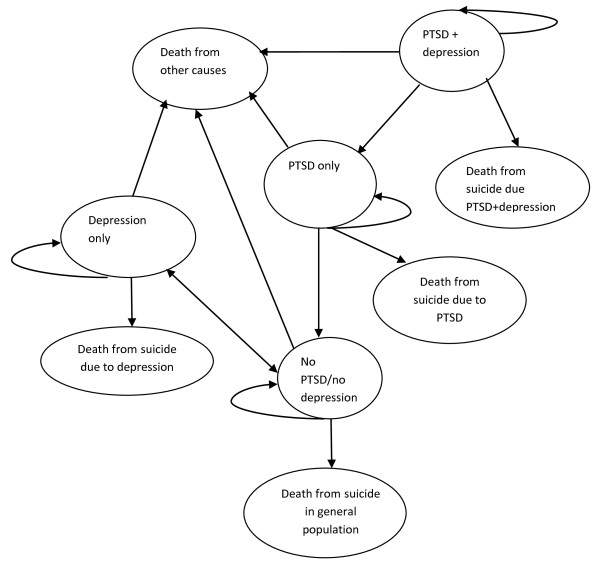
**Patient flow diagram for Markov model**.

In the Markov part of the model the cohort moves through mutually exclusive health states representing the possible mental health consequences associated with an experience of childhood sexual abuse. The model includes pathways for the gradual recovery from PTSD in those who did not respond to treatment and for recurrent-remitting depression (see below for further clarification). These are reflected as a set of possible transitions between the health states over a series of discrete time periods (cycles). The duration of the cycle in the model is 3 months with patients assumed to transition between states half-way through each cycle. Children and young adults who spend 3 months in any particular health state are assigned a utility value associated with this health state and may attract the cost of SSRI, if experiencing an episode of depression. The expected value of costs and QALYs are then calculated by adding the costs and health benefits across the states and weighting according to the time the person is expected to stay in each health state [41]. The structure of the Markov part of the model is shown in Figure 2.

### Pathways included in the economic model

The Markov model characterises disease process in terms of nine states (depicted as circles) and arrows indicating the transitions patients can make in the model. Responders to PTSD treatment start the progression through Markov cycles at the state of no PTSD/no depression. Non-responders start either at the PTSD only state or PTSD + depression if depression was present at the baseline. There are complex dynamics between PTSD and depression such that pre-existing depression increases susceptibility to developing PTSD in response to the traumatic event, while PTSD increases the risk for the first onset of depression [4].

However this Markov model, as all modelled economic evaluations, presents a simplified version of all possible variations of the lifetime history of PTSD with or without co-morbid depression. Because the objective is to evaluate treatments for PTSD secondary to childhood sexual abuse, any re-occurrence of PTSD due to subsequent traumatic life events is outside the scope of the evaluation. That implies that once PTSD is successfully treated there assumed to be no relapse associated with the original traumatic experience of sexual abuse. This is depicted in Figure 2 by a one-way arrow connecting the health state "PTSD only" with the health state "No PTSD/no depression".

While relapse to PTSD is not permitted in the model, the depression pathway is modelled differently; allowing for the recurrent-remitting nature of this mental health condition [42,43]. In Figure 2 the re-occurrence of depression in some children who responded to PTSD treatment and the subsequent recovery is depicted with a two-way arrow connecting the "No PTSD/no depression" state with the "Depression only" state. Children who at the baseline were diagnosed with both PTSD and depression but did not respond to treatment at the end of 12 months were subsequently administered SSRI as a second line treatment for depression as recommended by the clinical Guideline and existing practice [24,44]. The probability of successfully recovering from depression (obtained from the recent large RCTs) was applied to the proportion of patients who are compliant with the SSRI regimen [45]. This proportion of the cohort will progress to the "PTSD only" state. The model also assumes that a proportion of untreated children may experience a spontaneous recovery from depression, including those who may withdraw from the treatment because of side-effects. Those who responded to SSRI treatment for depression were assumed to continue on medication under supervision for the next 9 months after discontinuation of depression symptoms. This assumption is in line with evidence suggesting that longer medication continuation periods, possibly for one year, may be necessary for relapse prevention [46].

It should be noted that, consistent with the existing evidence, transition from the "PTSD + depression" state to "Depression only" is not allowed in the population of sexually abused children who are eligible for PTSD treatment (the baseline cohort). Firstly, it was demonstrated that the high rate of co-morbid depression in patients with PTSD is related to the same personal vulnerabilities. In other words, the hypothesis that traumatic life events increase the risk of depression independently of their PTSD effects was refuted [47]. Secondly, the existing evidence suggests that successful treatment of PTSD also results in remission of co-morbid depression with a 100% response rate in eligible patients [33,48]. However, eligibility for PTSD treatment may be compromised in patients with a co-morbid depression so severe that it makes PTSD treatment based on "exposure" methods impossible [20]. In such instances depression should be treated ahead of PTSD treatment. In either case, the positive treatment outcome that is limited to PTSD and not associated with greatly reduced symptoms of depression is unlikely.

The current evidence suggests that in comparison to the rates of suicides in the general population, children and adolescents suffering from PTSD or depression and especially from PTSD associated with co-morbid depression demonstrate higher rates of suicides [4,49-52]. The model includes age-related probabilities of suicide for each of the following health states: the PTSD + depression, PTSD only and Depression only to capture the long-term consequences of mental health interventions in terms of reduced rates of suicide.

According to the epidemiological evidence obtained for the purpose of this modelled economic evaluation, a proportion of children who did not respond to treatment for PTSD will eventually recover, although the process of recovery may take between two to 30 years. These estimates were obtained by the authors from an analysis of the 2007 Australian National Survey of Mental Health and Wellbeing data on duration of a PTSD episode believed to be associated with childhood sexual abuse [53]. Time-related transition probabilities from "PTSD only" to "No PTSD/no depression" were included in the model to reflect these respondents' (N = 124) time to recovery from PTSD onset. It was observed that in about one third of patients PTSD will persist for the rest of their lives. This is consistent with the estimated proportion of non-remitting PTSD patients observed in the USA population [38].

### Characteristics of the population included in the model

The demographic and clinical characteristics of the children included in the model cohort reflected the selection criteria of the RCTs that provided the estimates of clinical effectiveness for each of the evaluated interventions [15,16,33]. The baseline cohort consisted of 10-year-old children who met either all or most of the PTSD diagnostic criteria, including at least one symptom of avoidance or re-experiencing. Because PTSD often presents with delayed onset, the exclusion of children who did not meet full PTSD criteria at baseline was unwarranted [16]. For the purposes of economic evaluation the clinical outcomes were expressed in terms of the response rate (i.e. the proportion of children who were "PTSD-positive" at the baseline and "PTSD-negative" at post-treatment and the follow- up). Consequently, the children who did not meet the full PTSD diagnostic criteria at the baseline could not be considered responders at the post-treatment assessment either, although their trauma-related symptoms were improved [16]. The conservative definition of the "response rate" employed here resulted in an underestimate of clinical effectiveness of treatments and subsequently a conservative estimate of incremental cost-effectiveness ratios against the "no treatment" comparator. Children with psychiatric conditions that may be contraindicated to TF-CBT (e.g. severe developmental delay, psychosis, suicidal and dangerous or aggressive behaviour) were excluded. It was also required that any contact with an identified person involved in child sexual abuse had been discontinued.

### Data used in the model

The 2007 Australian National Survey of Mental Health and Wellbeing collected data from 8,841 participants aged 16 to 85 [53]. The survey included a generic preference-based instrument AQoL-4D for assessing health-related quality of life [39]. This particular version of AQoL used 12 scales to measure the interference that health problems in the week prior to the interview had on personal care, household tasks, ability to move around the house and community, personal relationships, relationships with other people; relationships with family, vision, hearing, communication with others, sleeping habits, feelings in general, and level of pain or discomfort. The responses to the AQoL-4D questionnaire by children and adolescents with a history of childhood sexual abuse who also met the DSM-IV diagnostic criteria for PTSD, PTSD + depression, or only depression were used to calculate utility estimates [54]. Utility values of 0.61 (SE = 0.08), 0.53 (SE = 0.09) and 0.46 (SE = 0.09) were used for the health states "PTSD only", "PTSD + depression" and "depression only" respectively for the entire time interval included in the modelled economic evaluation. The utility value of 0.87 observed in the 16-21 year old population without a history of sexual abuse was identical to the published AQoL-4D utility observed in the general population. This value was applied to the proportion of the cohort who either responded to treatment or experienced a spontaneous recovery over the age interval of 10-30. A slightly smaller value of 0.85 was used for the proportion of the cohort who remained in the "no PTSD/no depression" state when they were 30 to 40 years old [54].

Age-specific transition probabilities for the state "Death from other causes" were obtained from the Australian 2007-2009 Life Tables [52] and adjusted for the proportion of age-specific deaths from suicides in general population [37]. Suicide rates associated with other mental health conditions (Markov states "PTSD + depression", "PTSD only" and "Depression only") were obtained from the published literature [4,49-51].

Categories of mental health system resource use were obtained from the identified RCTs that provided clinical effectiveness estimates for the economic evaluation [15,16,33]. Resource use included the cost of therapists' time in providing 12 individual 45 minute TF-CBT or non-directive individual psychotherapy sessions per child in each of the active treatment arms. The cost of SSRI therapy (sertraline) was added to TF-CBT + SSRI treatment arm. Since psychotherapy can be provided by either psychologists or psychiatrists, it was assumed that each category of these mental health professionals treated one half of the cohort. The unit costs (scheduled fees) for psychologists and psychiatrists were taken from the MBS [55] and assumed to cover patient contact time, patient-related indirect time and overheads in publicly-funded youth mental health facilities. The cost of sertraline was taken from the Schedule of Pharmaceutical Benefits [56]. Since the PTSD Guideline are not specific about the form of non-abusive parent involvement in treatment, for the purpose of this study it was conservatively assumed that each parent received either one individual psychoeducational session with a social worker or participated in six parental group sessions. Cost implications are the same regardless of the modality of the parental involvement. Table 1 shows the model input parameters and the sources of the unit costs.

**Table 1:** Parameter values used in the model

Parameter	Value (range used in the deterministic sensitivity analysis)		Parameters used in probabilistic sensitivity analysis		Source
*Clinical effectiveness for treating PTSD (and depression if present). Used in the decision tree part of the model*			*Beta distribution*		
			α	β	
TF-CBT only	0.42 (0.29 - 0.55)*		24.3	33.6	[15,16,33]**
TF-CBT + SSRI (setraline)	0.44 (0.31 - 0.57)*		23.5	29.9	
Non-directive counselling	0.34 (0.24 - 0.44)*		27.8	54.0	
					
*Clinical effectiveness for treating recurrent depression (used in Markov part of the model) with SSRI*					
					
SSRI only (applied to treatment non-responders, and responders who relapsed into depression in the Markov part of the model)	0.6 (0.42 - 0.78)		16.5	11.0	[45,57]

*Disease pathways parameters*			*Beta distribution*		
			α	β	
Proportion of the cohort with co-morbid depression	0.58 (0.40 - 0.75)				[33]
					
Probability of delayed response to PTSD treatment (assumed to occur in the 6^th ^month after treatment)	0.17 (0.12 - 0.22)		35.3	172.1	[53]
					
Probability of spontaneous remission from PTSD (applied to the non- treated population in the first 12 months)	0.17 (0.12 - 0.22)		35.3	172.1	[18,53]
					
Probability of spontaneous recovery from PTSD over 29 years (applied to the treatment non-responders in Markov part of the model)	2 d year	0.0083			[53]***
	3-4th years	0.0041			
	5-6th years	0.0021			
	7-8th year	0.0043			
	9-11th year	0.0035			
	12-13th years	0.0023			
	14-16th years	0.0013			
	17-20th years	0.0028			
	21-29 years	0.0020			
					
Probability of spontaneous remission from depression	0.53 (0.37 - 0.69)		19.5	17.3	[45]
					
Probability of re-occurrence of depression in PTSD treatment responders	0.14 (0.09 - 0.18)		36.6	224.6	[33]

*Mortality*	0.0 (in 10-14 y.o.)				[37,58]
Age-adjusted probability of suicide in general population (per 3-month cycle)	0.0000155 (in 15-19 y.o.)				
	0.0000316 (in 20-29 y.o.)				
	0.0000387 (in 30-40 y.o)				
					
Age-adjusted probability of suicide in adolescents and young adults with PTSD + depression (per 3-month cycle)	0.0 (in 10-14 y.o.)				[37,49,58]
	0.000334 (in 15-19 y.o.)				
	0.000343 (in 20-29 y.o.)				
	0.000744 (in 30-40 y.o)				
					
Age-adjusted probability of suicide in adolescents and young adults with depression only (per 3-month cycle)	0.0 (in 10-14 y.o.)				[50]
	0.000186 (in 15-19 y.o.)				
	0.000379 (in 20-29 y.o.)				
	0.000465 (in 30-40 y.o)				
					
Age-adjusted probability of suicide in adolescents and young adults with PTSD only (per 3-month cycle)	0.0 (in 10-14 y.o.)				[51]
	0.000032 (in 15-19 y.o.)				
	0.000066(in 20-29 y.o.)				
	0.000080 (in 30-40 y.o)				
					
Age-adjusted probability of death from other causes except suicide in adolescents and young adults (per 3-month cycle)	0.000027 (in 10-14 y.o.)				[37,58]
	0.000070 (in 15-19 y.o.)				
	0.000104(in 20-29 y.o.)				
	0.000169 (in 30-40 y.o)				

*Mental health care resource use parameters*			*Gamma distribution*		
			α	λ	
One month prescription of sertraline, (100 mg)	$24.66		96.04	3.89	[56]
					
Cost per consultation with clinical psychologist/counsellor (MBS Australia Items 80000; 80010)	First consultation $140.90		96.04	0.68	[55]
	Subsequent consultation $96.00		96.04	1.00	
					
Cost per consultation with clinical psychiatrist (MBS Australia Items 296; 304)	First consultation $250.45		96.04	0.38	[55]
	Subsequent consultation $125.80		96.04	0.76	
					
Cost per consultation with general practitioner (monitoring and SSRI renewal if required)	$66.00		96.04	1.46	[59]
					
Cost of individual consultation with social worker or participation in 6 parental group sessions (Social workers schedule fee, Items SW01; SW15).	$58.85		96.04	1.63	[60]

*Utility values*					
			*Gamma distribution*		
No PTSD/No depression (population norm)			α	λ	
10-30 year olds	0.87				
30-40 year olds	0.85				[53,54]
PTSD only	0.61 (0.43 - 0.79)***		96.04	157.4	
PTSD + depression	0.53 (0.37 - 0.69)***		96.044	181.21	
Depression only	0.46 (0.32 - 0.60)***		96.044	208.78	

*The aggregated sample size used in direct and indirect comparison of effectiveness of active treatments vs no treatment comparator was: N = 50 in TF-CBT [16]; N = 36 in TF-CBT + sertraline [16,33]; N = 116 in non-directive counselling [15,16]

**Clinical effectiveness (proportion of responders at the end of treatment) was adjusted for indirect comparison;

***Estimate obtained by the first author. (E. Gospodarevskaya "Prevalence of post-traumatic stress disorder, symptom duration and quality of life in sexually abused Australian children: using mental health survey data for economic analysis"; Journal of Child Sexual Abuse, 2012. *Forthcoming*)

Results of the modelled economic evaluation are presented in terms of incremental cost per QALY gained. Results are presented separately for the 12 month time interval that corresponds to the follow-up outcomes reported in RCTs and for the long-term outcomes with a time horizon of 31 years. The economic evaluation is conducted from the perspective of the Australian mental health care system; costs and benefits are expressed in 2010/2011 Australian dollars and discounted at a rate of 5% per year.

### Uncertainty

All model parameters other than unit costs and population utility norms were subjected to deterministic and probabilistic sensitivity analyses. For some parameter estimates (e.g. probabilities of spontaneous remission, reoccurrence of depression and suicide rates) no measure of variability was available from the epidemiological evidence. In the sensitivity analyses an arbitrarily chosen 30% variation range around each of the parameter point estimates was used (i.e. the modelled results were recalculated for 70% and then for 130% of each point estimate as shown in Table 1). Two-way sensitivity analysis for TF-CBT and TF-CBT + SSRI clinical effectiveness parameters was undertaken (i.e. the lower and upper values were assigned to both treatment alternatives) to account for the possible covariance between these two treatment options because it was reasonable to assume that effectiveness of TF-CBT is a component of effectiveness of TF-CBT + SSRI. Utility estimates were obtained by the first author from the Australian 2007 Mental Health Survey [53], which allowed testing the robustness of the outcomes to variation in these model parameters using firstly, the 30% sensitivity range and secondly, the 95% confidence intervals. The small sample of the population available for calculating utility values in sexually abused children who developed adverse mental health consequences resulted in the counter-intuitive point estimates where utility associated with depression only (0.46; N = 11) was less than utility associated with PTSD + depression (0.53; N = 9), although the difference was not statistically significant indicating that variation in the estimates is likely due to the randomness of the data. To investigate the effect of variation in utility estimates we conducted a two-way sensitivity analysis using the higher (0.53) and the lower (0.46) point estimates for both utility values.

In addition, a probabilistic sensitivity analysis was conducted. Firstly, the parameter estimates other than population-based utility norms and suicide rates were assigned a probability distribution as shown in Table 1. Secondly, Monte-Carlo simulation was used to reflect the uncertainty in the model's results and calculate 95%CI around estimates of costs and QALYs [41].

## Results

Table 2 shows results of cost-effectiveness analysis with a time horizon of 12 months (the decision tree part of the model). Using the no-treatment option as a comparator, the observed difference in clinical effectiveness translated into an incremental QALY gain ranging from 0.06 in non-directive supportive counselling to 1.0 in TF-CBT + SSRI. The estimated ICERs of active treatment vs. no treatment range from A$22,263 for TF-CBT + SSRI to A$34,567 for non-directive counselling, indicating that even in the short term investing in any type of psychotherapy is likely to present a good value for money from the perspective of the Australian mental health system if the threshold of A$50,000 per QALY gained is considered 'affordable' [61].

**Table 2:** Results of the 12 month decision tree analysis

Treatment options	Total cost per child (A$2010-2011)	Total QALYs	Incremental QALYs vs no treatment	Incremental cost per QALY gained vs no treatment	Incremental cost per QALY gained (comparing to non-dominated treatments)
No treatment	0	0.87	-	-	-
Non-directive counselling	2074.0	0.93	0.06	34,567	Dominated by TF-CBT
TF-CBT only	2051.1	0.96	0.09	22,790	(2226.3-2051.1)/(0.97-0.96) = 17,520
TF-CBT + SSRI (sertraline)	2226.3	0.97	0.10	22,263	

The Markov model with a 30-year time horizon was designed to trace down the long-term costs associated with recurrent-remittent depression and the benefits associated with an improved quality of life and reduced rates of suicides in treatment responders. As explained in the Methods section, the model is limited to its objective of evaluating alternative therapies for treatment of PTSD secondary to childhood sexual abuse. Consistent with its objective, any costs associated with any subsequent PTSD related to other traumatic events are not included in the model. The prognostic model effectively translates benefits of treatment (QALYs gained) accrued during the initial 12 month interval into differences in long-term costs and QALYs. In the long-term cost-effectiveness analysis the discounted benefit of QALY gains associated with a reduction in suicide rates that would otherwise increase in 10 to 20 years after PTSD treatment was smaller than the accumulated effect associated with the QALY gain obtained by the treatment responders at 12 months. Table 3 shows the results of the base-case analysis of the long-term Markov model. The estimated ICERs of active treatments vs. no treatment range from just over A$1,650 for TF-CBT only to under A$2,100 for non-directive counselling.

**Table 3:** Results of the base-case analysis of the model with the 31 year time horizon

Treatment options	Total cost per child (A$2010-2011)	Total QALYs	Incremental QALYs vs no treatment	Incremental cost per QALY gained vs no treatment	Incremental cost per QALY gained (comparing to non-dominated treatments)
No treatment	0	11.59	-	-	-
Non-directive counselling	2123.2	12.61	1.02	2081.57	Dominated by
TF-CBT only	2095.7	12.86	1.28	1650.16	(2269.8-2095.7)/(12.92-12.86) = 2901.7
TF-CBT + SSRI (sertraline)	2269.8	12.92	1.34	1706.61	

The Guideline for Management of PTSD in Adults and Children [20] recommended TF-CBT over the non-directive counselling. Consistent with this recommendation, results of both the short- and long-term modelled economic evaluation indicated that TF-CBT generated more QALYs and cost less than non-directive supportive counselling (i.e. dominating this treatment option). The combination therapy of TF-CBT and SSRI generated more QALYs than either non-directive counselling or TF-CBT only options. However, quite predictably, the combination therapy was more expensive than TF-CBT alone in either the short- or long-term versions of the model.

Extensive one-way sensitivity analyses were conducted by varying the model parameters as indicated in Table 1. The primary objective of the analysis was to identify the parameter values associated with the ICER exceeding the A$50,000 threshold. The secondary objective of the sensitivity analysis was to determine the parameter values that change the order of preference in the active treatments established in the base-case analysis.

Results were robust with respect to variation in most parameters of the model (e.g. rates of suicides, probability of spontaneous remission from PTSD, proportion of cohort with co-morbid depression, probability of delayed response to PTSD treatment, effectiveness of SSRI for treatment of depression and health state specific utility estimates). The results of these sensitivity analyses showed that non-directive supportive counselling remained more costly and less effective than the TF-CBT treatment. The TF-CBT + SSRI treatment remained the most effective but also the most expensive of the active treatment alternatives. In each case the ICERs for these preferred treatment options remained below A$2,000 using a no treatment alternative as a comparator. The only exception was ICER estimates for the upper limit of the utility estimate for PTSD (0.79). The ICER values were A$6,513 for TF-CBT and A$6,617 for TF-CBT + SSRI, with non-directive counselling dominated by these treatment options. When the sensitivity analysis was replicated using the 95% CI for utility estimates, the results changed very little because the 30% parameter variation range was slightly larger than the 95% CI. Conducting a two-way sensitivity analysis with both utility values for PTSD + depression and depression only assigned firstly the value of 0.53 and then the value of 0.46, produced only a small variation in the results of the base-case analysis and did not affect the overall conclusions.

At the upper limit of the probability of successful PTSD treatment with non-directive counselling (0.44), this treatment option dominated both TF-CBT and TF-CBT + SSRI treatment options. Under these assumptions TF-CBT and TF-CBT + SSRI generated about the same number of QALYs but were marginally more expensive than non-directive counselling with the ICERs of A$1,650 and A$1,706 respectively. When the clinical effectiveness of both TF-CBT and TF-CBT + SSRI was held at the lower level of 0.29 and 0.31 respectively, TF-CBT produced 12.45 QALYs at an additional cost of A$2,140, while TF-CBT + SSRI generated slightly more QALYs (12.51) at a marginally higher cost of A$2,314. Under these assumptions, non-directive counselling again dominated both TF-CBT and TF-CBT + SSRI. The ICERs of TF-CBT and TF-CBT + SSRI vs no treatment were A$2,489 and A$2,516 respectively. The ICER of TF-CBT + SSRI vs TF-CBT was A$2,902. Conversely, when clinical effectiveness of both TF-CBT and TF-CBT + SSRI was held at the upper level of 0.57 and 0.55 respectively, non-directive counselling was dominated by TF-CBT. The results of this two-way sensitivity analysis were 13.27 QALYs at an additional cost of A$1,221 for TF-CBT; 13.34 QALYs at an additional cost of A$1,271 for TF-CBT + SSRI as compared to 12.61 QALYs at an additional cost of A$2,081 for non-directive counselling.

Results of probabilistic sensitivity analysis including 95%CI around costs and QALY outcomes are shown in Table 4.

**Table 4:** Results of probabilistic sensitivity analysis of the long-term model (1000 trials)

Treatment options	Mean cost per child in A$2010-2011 (95%CI)	Mean number of QALYs (95%CI)	Mean incremental number of QALYs vs no treatment	ICER
No treatment	0	11.57	-	-
		(10.24; 13.06)		
Non-directive counselling	2125	12.59	1.02	2083
	(1927; 2335)	(11.62; 13.57)		
TF-CBT only	2097	12.85	1.28	1638
	(1904; 2302)	(11.98; 13.74)		
TF-CBT + SSRI (sertraline)	2271	12.92	1.35	1682
	(2070; 2478)	(12.01; 13.78)		

While the mean values of costs and outcomes closely approximate the point estimates obtained in the deterministic base-case analysis reported in Table 3, the size of the confidence intervals reflect the degree of uncertainty around the parameter estimates included in the model.

## Discussion

The study adds to current knowledge about comparative cost-effectiveness of different treatment options in the population of sexually abused children and adolescents. Governments in high-income countries increasingly rely on economic evidence, preferably in the form of cost-utility analysis [62,63], to inform decisions about funding of health services. Unlike NICE in the UK, Australian funding bodies do not use a fixed threshold in its decision making. For example, past PBAC decisions suggest the use of a background willingness to pay for health gains that varies with the opportunity cost of the proposed drug [64]. However, the listing of pharmaceuticals on the PBS and receipt of considerable government subsidy are more likely for interventions with ICERs below A$50,000 per QALY gained [65]. In the base-case and sensitivity analyses, ICERs for all three active treatment alternatives were always less than A$7,000 per QALY gained indicating that, when compared to no treatment, each of these interventions would likely be considered a good investment from the perspective of the Australian mental health system.

Results are more equivocal for the comparison between active treatments. The base-case results indicated that non-directive counselling is dominated by other active treatments: TF-CBT and TF-CBT + SSRI, and that efficiency gain (more QALYs for a given investment) can be achieved by allocating more resources towards these therapies. However, this result was sensitive to variation in the clinical effectiveness parameters with non-directive counselling dominating TF-CBT and TF-CBT + SSRI under certain assumptions. Moreover, while the best available evidence suggests that TF-CBT + SSRI is more effective (and more cost-effective) than TF-CBT alone, there is a continuing debate about risks of suicidal behaviour/ideations associated with prescribing SSRIs to children and adolescents [31,66]. The limited clinical evidence used in the present study did not produce any support for the elevated risk of suicidal behaviour/ideations in the population of children and adolescents treated for PTSD secondary to sexual abuse with a combination therapy of TF-CBT + SSRI [33]. Therefore the outcomes of the modelled economic evaluation were not adjusted for the adverse risk profile believed to be associated with SSRI prescribing. However, it should be noted that this also implies that results of the present study are not generalisable beyond the population who met the inclusion criteria used in the clinical trials that informed the economic evaluation. In particular, if children demonstrated suicidal behaviour or harm to others at the pre-treatment assessment they would become ineligible for TF-CBT with or without SSRI. In such instances the PTSD Guideline suggests that healthcare professionals should first concentrate on management of these risks [20].

Although the evidence of the increased adolescent mortality due to suicides believed to be associated with SSRI prescribing is inconclusive [46] it is possible that even with respect to children who did not experience suicidal behaviour/ideations at the pre-treatment assessment some clinicians and parents may exercise caution when deciding on the best treatment option, and the maximum health benefit associated with TF-CBT + SSRI will not be achieved. Given uncertainty associated with clinical effectiveness of the evaluated interventions, it may not always be possible to achieve an optimal decision in resource allocation where implementation of that decision relies upon changes in prescribing practice and patient preferences.

There is a scarcity of cost-utility studies in the paediatric population and particularly in the area of child mental health. In the absence of prospective epidemiological studies in the population of sexually abused children, the modelled evaluations rely on a number of assumptions that remain to be validated against future research, although these are always legally and ethically challenging. The economic evaluation reported here was undertaken from a limited clinical perspective and does not include the long-term social and economic implications (employments, sexual health, educational achievements) that would be required if a societal perspective was undertaken. Currently there is no evidence that would allow drawing an association between the degree of success of different PTSD treatments and other socio-economic factors that influence wellbeing of children and adolescents.

In all modelled economic evaluations the validity of results depends on the quality of data available and the simplifying assumptions about progression of disease over the modelled time horizon. For this model, clinical effectiveness parameter estimates were obtained from high quality RCTs, where reduction in PTSD symptoms was the primary outcome. The outcomes of economic evaluation therefore apply only to the comparative analysis of treatments used for trauma-related problems in sexually abused children. All but one [33] of these RCTs were used as an evidential basis in the Clinical Practice Guideline for Management of PTSD in Adults and Children [20]. The limited number of treatment alternatives modelled reflects the lack of quality trials of other therapies, e.g. child-parent psychotherapy, parent-child interaction therapy and structured group treatments [20,67].

Since there are no published utility estimates associated with the health states included in the model, health-state utility values and age-specific probabilities of gradual recovery from PTSD over the modelled time interval were obtained from the 2007 Australian National Survey of Mental Health and Wellbeing [53]. Although the Mental Health Survey included a representative sample of the Australian population, the number of young people aged 16-21 who reported a history of childhood sexual abuse was limited to 82 respondents and only a proportion of these respondents met DSM diagnostic criteria for PTSD with or without depression. Children younger than 16 years old were not included in the survey necessitating assignment of utility estimates obtained from the adolescent population to the model cohort of children (10 years old at the baseline). Nevertheless sensitivity analysis on utility estimates did not produce outcomes that would invalidate the results of the base-case analysis.

One of the strong assumptions of the model is that the difference in effectiveness between the treatments only relates to the first 12 months for which the post-treatment and follow-up effectiveness outcomes are available. There is no evidence to suggest that the likelihood of recurrent depression or the propensity to seek additional treatment in non-responders is influenced by the type of initial treatment. In the absence of any supporting evidence on the differential distribution of the subsequent mental health conditions and the patterns of health care resource use, it may be reasonable to suggest that the more effective initial treatment will generate additional savings by preventing a higher proportion of the original cohort from seeking assistance for their mental health problems in the future. From this point of view, the "prognostic" model that extends results observed at the 12 month follow-up over the next 30 years will generate a conservative ICER estimate.

It should also be noted that the existing clinical evidence is limited to the first line of PTSD treatment in sexually abused children. There is neither a clinical guidance on the secondary treatment options for non-responders nor reliable data on existing clinical practices. Therefore, the model is limited to the first line PTSD treatment options, while subsequent health care resource use relates only to the episodes of co-morbid depression. While international cost-of-illness studies suggest that PTSD is associated with a considerable increase in health care resource use (around US$4,500 in 2005 prices adjusted for purchasing power parity [68]), the direct medical cost data should be balanced against the evidence of clinical effectiveness and ideally reflect clinical pathways specific to PTSD secondary to childhood sexual abuse.

In relation to the first line treatment, there is some evidence that TF-CBT is under-represented in the current mix of treatments. For example, a preliminary analysis of the 2007 Australian Mental Health Survey data showed that out of 400 respondents who had met DSM-IV criteria for PTSD in the last 12 month, only 18% reported that they received CBT. Although respondents may be lacking in their ability to accurately identify the type of received treatment, the results are consistent with the results of a survey of 852 psychologists in the USA, where only 17% stated that they use exposure therapy for PTSD [69]. Whereas the present study estimates the relative cost-effectiveness of alternative interventions, policy decisions regarding active implementation of the Guideline recommendations would require estimation of the net benefits associated with moving from current practice patterns to the practice patterns recommended by the PTSD Guideline. Based on the results of the economic evaluation presented here, any treatment mix associated with a higher proportion of children receiving TF-CBT and TF-CBT + SSRI (as recommended by the PTSD Guideline) is likely to be more cost-effective than the current mix of treatments. This may raise a policy concern regarding the availability of suitably trained psychologists and psychiatrists and the funding of their services.

## Conclusion

Even after accounting for uncertainty in parameter estimates, the results of the modelled economic evaluation demonstrated that under Australian health services conditions all active psychotherapy treatments for PTSD in sexually abused children have a favourable ICER relative to no treatment. The results also highlighted the loss of quality of life in children who do not receive any psychotherapy. Results are more equivocal for the comparison between active treatments. The base-case results indicated that non-directive counselling is dominated by other active treatments: TF-CBT and TF-CBT + SSRI, and that efficiency gain (more QALYs for a given investment) can be achieved by allocating more resources towards these therapies. On the basis of the best clinical evidence, it is apparent that TF-CBT + SSRI is more cost-effective than TF-CBT alone, however, considering the uncertainty associated with prescribing SSRIs to children and adolescents, clinicians and parents may exercise some caution in choosing this treatment alternative, which may result in suboptimal allocation of the scarce mental health services resource.

## Abbreviations

AQoL: Assessment of Quality of Life; CI: confidence interval; DSM: Diagnostic and Statistical Manual of Mental Health Disorders; EQ-5D: European Quality of Life five dimensions; ICER: Incremental Cost-Effectiveness Ratio; MBS: Medicare Benefits Schedule; NICE: National Institute for Health and Clinical Excellence; PBAC: Pharmaceutical Benefits Advisory Committee; PBS: Pharmaceutical Benefits Schedule; PTSD: Post-traumatic stress disorder; QALY: Quality Adjusted Life Year; RCT: Randomised controlled trial; SE: standard error; SSRI: Selective Serotonin Reuptake Inhibitor; TF-CBT: Trauma-Focused Cognitive Behavioural Therapy; 95%CI: 95% confidence interval.

## Competing interests

The research was funded by the Australian Research Council (ARC) Linkage Grant (LP0883743). The ARC is a statutory authority with the mission to deliver policy and programs that advance Australian research and innovation globally and benefit the community. Linkage Projects supports research and development projects which are collaborative between higher education researchers and other parts of the national innovation system, which are undertaken to acquire new knowledge, and which involve risk or innovation. The authors declare that the organisational partners of the ARC grant did not influence the results presented in the manuscript.

## Authors' contributions

EG conceived and designed the study, acquired the data, designed and reviewed the model, analysed and interpreted the data, drafted the manuscript and gave final approval of the version to be published. LS obtained funding for the study, reviewed the draft of the manuscript and gave final approval of the version to be published. Both authors read and approved the final manuscript.
